# Olfactory perceptual decision-making is biased by motivational state

**DOI:** 10.1371/journal.pbio.3001374

**Published:** 2021-08-26

**Authors:** Laura K. Shanahan, Surabhi Bhutani, Thorsten Kahnt

**Affiliations:** 1 Department of Neurology, Feinberg School of Medicine, Northwestern University, Chicago, Illinois, United States of America; 2 School of Exercise and Nutritional Sciences, College of Health and Human Services, San Diego State University, San Diego, California, United States of America; 3 Department of Psychiatry and Behavioral Sciences, Feinberg School of Medicine, Northwestern University, Chicago, Illinois, United States of America; 4 Department of Psychology, Weinberg College of Arts and Sciences, Northwestern University, Evanston, Illinois, United States of America; University of Cambridge, UNITED KINGDOM

## Abstract

Growing evidence suggests that internal factors influence how we perceive the world. However, it remains unclear whether and how motivational states, such as hunger and satiety, regulate perceptual decision-making in the olfactory domain. Here, we developed a novel behavioral task involving mixtures of food and nonfood odors (i.e., cinnamon bun and cedar; pizza and pine) to assess olfactory perceptual decision-making in humans. Participants completed the task before and after eating a meal that matched one of the food odors, allowing us to compare perception of meal-matched and non-matched odors across fasted and sated states. We found that participants were less likely to perceive meal-matched, but not non-matched, odors as food dominant in the sated state. Moreover, functional magnetic resonance imaging (fMRI) data revealed neural changes that paralleled these behavioral effects. Namely, odor-evoked fMRI responses in olfactory/limbic brain regions were altered after the meal, such that neural patterns for meal-matched odor pairs were less discriminable and less food-like than their non-matched counterparts. Our findings demonstrate that olfactory perceptual decision-making is biased by motivational state in an odor-specific manner and highlight a potential brain mechanism underlying this adaptive behavior.

## Abstract 2

Growing evidence suggests that internal factors influence how we perceive the world; this study shows that food intake influences how humans perceive food odors, such as the scent of cinnamon buns. This effect is specific to meal-matched odors, and is paralleled by changes in odor-evoked brain activity.

## Introduction

Sensory perception is typically thought to reflect physical reality, but closer examination often thwarts this notion. In fact, the way we perceive the world can depend on various motivational factors. Evidence for such “motivated perception” has been shown in the visual domain, where human participants are more likely to perceive cues that are motivationally salient [1–4]. The sense of smell may be particularly susceptible to such influences. The olfactory system shares substantial anatomical overlap with the limbic system [5], and prior work suggests that odor perception and its neural correlates are remarkably flexible [6,7]. For instance, odor percepts can be shaped by associative learning [8–10], expectations [11], and selective attention [12,13].

Given the intimate link between olfaction and food intake, odor perception may also be prone to influences by hunger and satiety. Indeed, previous studies have shown that the subjective value of food odors decreases with food intake [14–21] and that satiety and fluctuations in appetite-regulating hormones might influence olfactory sensitivity [22–26]. However, human work addressing this question relies heavily on subjective pleasantness ratings and threshold testing for single odorants. Whether hunger and satiety modulate olfactory perceptual decision-making has not been examined.

Perceptual decision-making in the visual domain has been widely studied using motion discrimination tasks, where subjects view moving dots and report their dominant direction [27–30]. Experiments employing simple tasks like these have yielded important insights into the neural basis of decision-making, but little is known about perceptual decisions in the olfactory domain. Prior work in this area is limited to a number of animal studies [31,32] and one human study [33], and the neural substrates through which food intake might modulate olfactory perceptual decisions in humans are not known.

There are 2 possible ways in which neural processing of olfactory stimuli could adapt to accommodate state-dependent changes. First, changes could occur directly in primary olfactory and limbic brain regions like olfactory bulb, piriform cortex, and amygdala [34–37], such that rudimentary stimulus representations adjust to reflect state-dependent goals. Supporting this idea, previous work in rodents has demonstrated that fasting can influence signaling at the level of the olfactory bulb [38–41]. Along similar lines, partial sleep deprivation can sharpen odor representations in human piriform cortex [42], suggesting that state-dependent modulation may occur early on in olfactory pathways. Alternatively, satiety state could impact neural activity in higher-order brain areas, leaving early sensory representations intact. The insular cortex responds to food-related stimuli across sensory modalities [43–47] and is thus a promising candidate for modulating neural processing of olfactory stimuli in a state-dependent manner.

Here, we designed a novel behavioral task involving binary food and nonfood odor mixtures to test whether motivational states (fasted versus sated) influence olfactory perceptual decision-making in humans. Moreover, we used functional magnetic resonance imaging (fMRI) to investigate the neural substrates governing such state-dependent changes. We found that food intake biased perceptual choices regarding food odors, such that participants were less likely to perceive the same odor mixture as food dominant following an odor-matched meal. Pattern-based fMRI analyses revealed that these behavioral effects were paralleled by neural changes in odor-specific representations in olfactory/limbic brain areas.

## Results

### A novel behavioral task to probe olfactory perceptual decision-making

**Fig 1:**
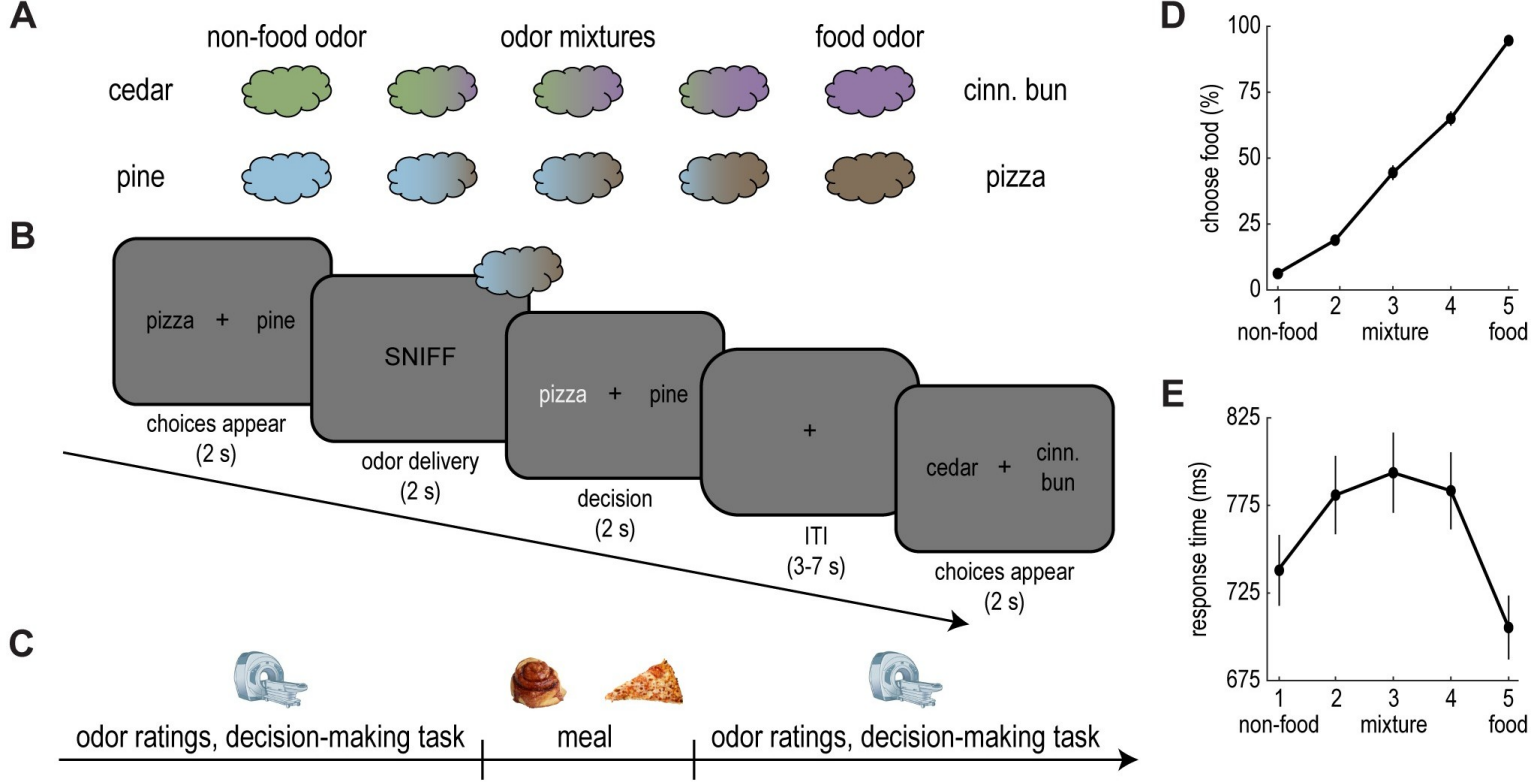
A novel behavioral task to probe olfactory perceptual decision-making. **(A)** Odor stimuli consisted of 4 pure odors (2 food and 2 nonfood) and 6 binary odor mixtures combining food and nonfood components. **(B)** Example trial. After choice labels appeared, participants received an odor and selected the component they perceived as dominant. Label position was counterbalanced across trials. **(C)** Experimental timeline. Participants completed the perceptual decision-making task during fMRI scanning, both before and after a meal. In addition, participants made appetite and odor ratings pre- and post-meal. **(D)** Perceptual choices. Participants were more likely to perceive odors as food dominant as the concentration of the food component increased. **(E)** Response times. Perceptual choices were slower for the odor mixtures. Error bars depict SEM for *n* = 30. Individual participant data summarized in these plots can be found in **S1 Data**. fMRI, functional magnetic resonance imaging; ITI, intertrial interval; SEM, standard error of the mean.

We designed a new behavioral task to measure the influence of motivational state on olfactory perceptual decision-making. In this task, human participants (*n* = 30) were exposed to binary odor mixtures containing food and nonfood components (i.e., cinnamon bun and cedar; pizza and pine). The stimulus set consisted of 10 unique mixtures in total, 5 per odor pair (**Fig 1A**). Mixtures varied in terms of concentration, ranging from the pure food odor to the pure nonfood odor. On each trial, participants were presented with an odor mixture, and they had to decide whether the food or nonfood component was dominant in the mixture (**Fig 1B**). Specifically, depending on the trial type, participants chose between cinnamon bun and cedar or pizza and pine.

Participants arrived hungry (fasted at least 6 hours), and they completed the perceptual decision-making task during fMRI acquisition both before and after an experimental meal (**Fig 1C**). This way, we could compare task performance and neural activity across fasted and sated states. Critically, the interim meal was matched to 1 of the 2 food odors and consisted of either cinnamon buns (*n* = 15) or pizza (*n* = 15), and participants were instructed to eat as much as they could until they were very full. By collapsing across meal groups, we could test the specific effect of food intake on the perception of meal-matched and non-matched food odors, regardless of meal identity. For instance, for participants who received cinnamon buns, the meal-matched odor pair was cinnamon bun and cedar, while the non-matched odor pair was pizza and pine. Note that, in addition to consuming the meal, participants were exposed to the sight, smell, and taste of the food. Although we cannot completely rule out the possible contribution of such factors, prior work suggests that perceptual changes in this context depend on ingestion rather than mere food exposure [48–50].

As expected, participants were more likely to perceive odor mixtures as food dominant as the relative concentration of the food component increased (**Fig 1D**). Moreover, in line with perceptual decision-making in other sensory domains [29,30,51], the average response time across mixtures followed an inverted U shape (**Fig 1E**). That is, perceptual choices were faster for pure odors compared to the more ambiguous odor mixtures.

Participants also made appetite and odor ratings before and after the meal. Appetite ratings indicated that the meal manipulation was successful in inducing satiety, as participants reported a significant decrease in hunger from immediately before to immediately after the meal (t_(29)_ = 19.31, *p* < 0.001; **S1 Fig**). Moreover, participants rated pure food odors as less pleasant after the meal compared to beforehand (t_(29)_ = 8.19, *p* < 0.001; **S2A Fig**), which aligns well with prior work [14–20]. This decline in pleasantness ratings was observed for both meal-matched and non-matched food odors (meal-matched: t_(29)_ = 6.43, *p* < 0.001; non-matched: t_(29)_ = 5.15, *p* < 0.001; **S2A Fig**). Finally, ratings of intensity and food-likeness were also influenced by the meal (**S2B and S2C Fig)**.

### Satiety biases olfactory perceptual decision-making for meal-matched odors

**Fig 2:**
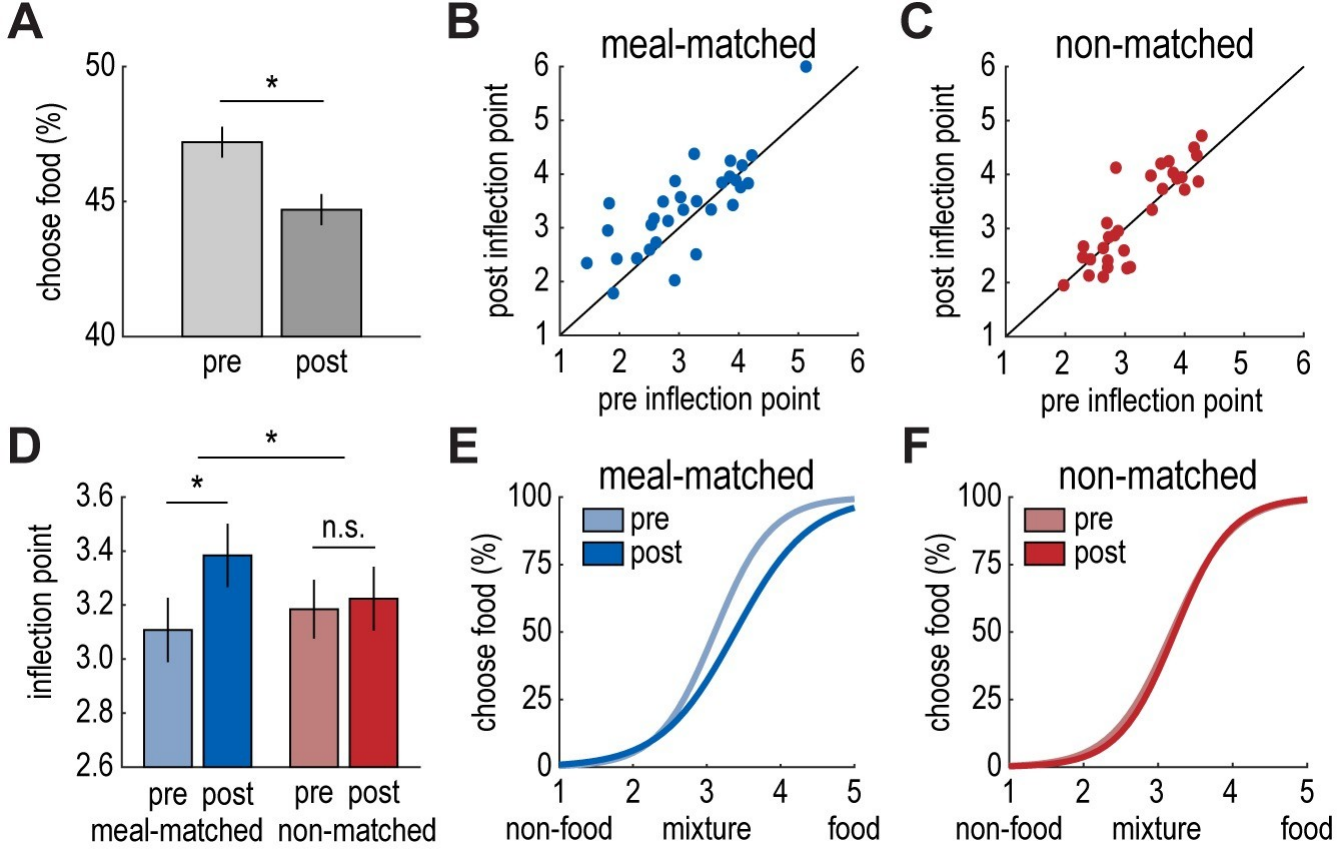
Satiety biases olfactory perceptual decision-making for meal-matched odors. **(A)** Participants were less likely to identify the food component as dominant in the mixture following the meal (t_(29)_ = 2.19, *p* = 0.04). **(B, C)** Individual participant data depicting the inflection point of the sigmoidal function pre- and post-meal for the meal-matched (B) and non-matched (C) odor pairs. **(D)** The sigmoidal inflection point increased significantly from before to after the meal for the meal-matched odor pair (t_(29)_ = 2.66, *p* = 0.01), indicating a bias away from the food odor. This was not the case for the non-matched odor pair (t_(29)_ = 0.49, *p* = 0.63). Inflection points increased more for the meal-matched odor pair compared to the non-matched odor pair (t_(29)_ = 1.92, *p* = 0.03, 1-tailed). **(E, F)** Perceptual choice curves (with parameters reflecting the mean inflection point and slope across participants) illustrating the average shift in the inflection point from pre- to post-meal for meal-matched (E) and non-matched (F) odor pairs. Error bars depict within-subject SEM for *n* = 30. Individual participant data summarized in these plots can be found in **S1 Data**. SEM, standard error of the mean.

Next, we measured participants’ olfactory perceptual choices across fasted and sated states. We first compared the percentage of trials where participants perceived the food component (i.e., cinnamon bun or pizza) as dominant in the mixture across pre- and post-meal sessions. Interestingly, participants were less likely to perceive the food component as dominant during the post-meal session (t_(29)_ = 2.19, *p* = 0.04; **Fig 2A**). We observed a similar trend (t_(15)_ = 1.64, *p* = 0.12) in a smaller pilot sample (*n* = 16) that completed the full experiment without MRI.

To gain a finer-grained understanding of how an odor-matched meal impacts perceptual decision-making, we applied psychometric modeling to perceptual choice behavior across mixtures on a participant-by-participant basis. Specifically, we fit a sigmoidal function to each participant’s perceptual choices across odor mixtures for each odor pair and task session, resulting in 4 choice curves per participant (**S3 and S4 Figs**). This allowed us to measure shifts in perceptual choices from pre- to post-meal for meal-matched and non-matched odor pairs separately. We predicted that choice curves would shift toward the pure food odor after the meal, indicating that a larger proportion of the food component would be required for participants to perceive mixtures as food dominant. That is, we anticipated a post-meal increase in the inflection point of the sigmoidal function (i.e., the food/nonfood ratio at which participants were equally likely to classify the mixture as food or nonfood dominant), which would reflect a bias toward the nonfood odor.

Interestingly, we observed the expected shift for the meal-matched odor pair (t_(29)_ = 2.66, *p* = 0.01; **Fig 2B and 2D**), but not for the non-matched odor pair (t_(29)_ = 0.49, *p* = 0.63; **Fig 2C and 2D**). Moreover, the increase in the inflection point from pre- to post-meal was larger for the meal-matched odor pair compared to the non-matched odor pair (t_(29)_ = 1.92, *p* = 0.03, 1-tailed; **Fig 2D**), but did not differ by sex (t_(28)_ = 0.93, *p* = 0.36). This finding suggests that food intake alters perceptual decision-making by biasing choices away from odors that match recently consumed foods. In contrast, we did not observe a significant change in the slope of the sigmoidal functions for either odor pair from before to after the meal (meal-matched: t_(29)_ = 1.46, *p* = 0.15; non-matched: t_(29)_ = 0.42, *p* = 0.68; time by odor pair interaction: t_(29)_ = 1.32, *p* = 0.20; **S5A–S5C Fig**), suggesting that the experimental meal did not induce broader changes in olfactory perceptual acuity. Rather, food intake seems to induce a relative bias away from the food odor in a meal-specific manner (**Fig 2E and 2F**).

### fMRI ensemble patterns in olfactory/limbic areas and insula discriminate food and nonfood odors

**Fig 3:**
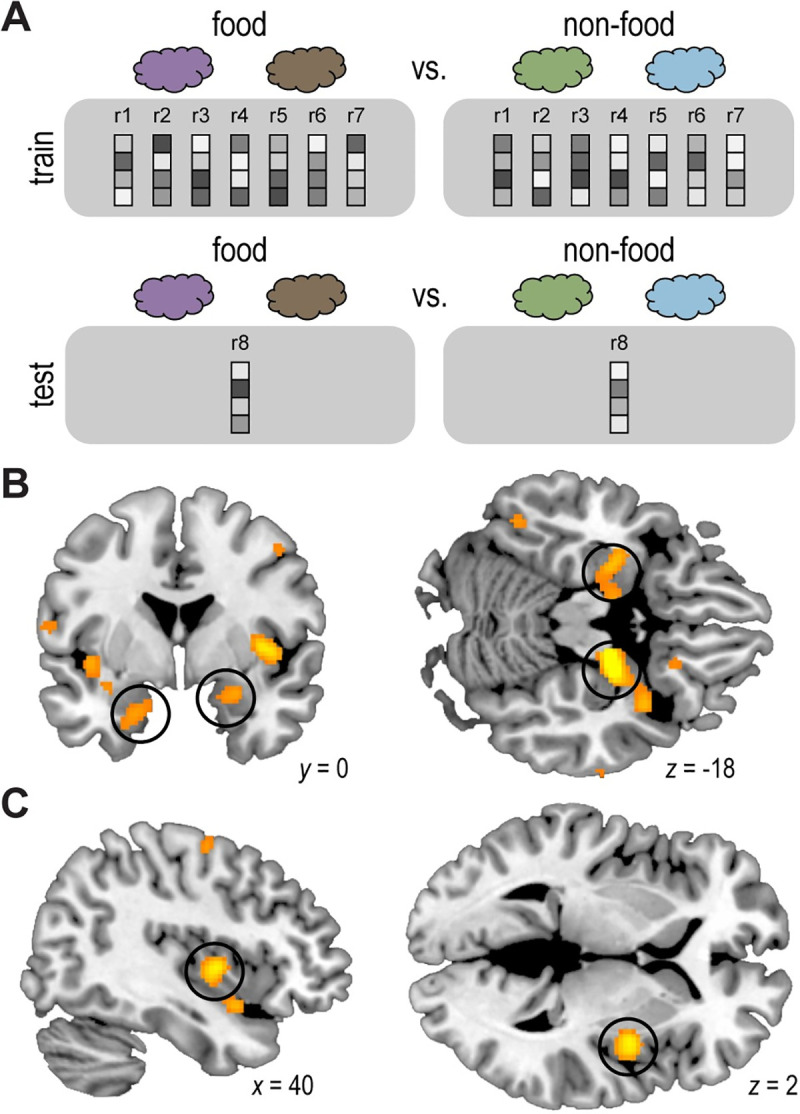
fMRI ensemble patterns in olfactory/limbic areas and insula discriminate food and nonfood odors. **(A)** Schematic of searchlight decoding analysis. For each searchlight sphere, an SVM classifier was trained and tested on ensemble patterns of fMRI activity evoked by pure food (average of cinnamon bun and pizza) vs. nonfood (average of cedar and pine) odors during both pre- and post-meal sessions using a leave-one-run-out cross-validation approach. **(B, C)** Significant decoding accuracy reflecting food vs. nonfood pattern discrimination in bilateral olfactory/limbic brain areas (left: x = −28, y = −6, z = −18, t_(29)_ = 6.81, *p*_*FWE*_ = 0.003; right: x = 20, y = −8, z = −18, t_(29)_ = 10.11, *p*_*FWE*_ < 0.001; B; circled) and right insula (x = 40, y = −2, z = 2, t_(29)_ = 7.85, *p*_*FWE*_ < 0.001; C; circled). The group-level t-map is thresholded at *p*_*FWE*_ < 0.05 and overlaid on a canonical structural image. The whole-brain statistical map can be viewed at neurovault.org/collections/EWYJXOKG/images/510268/. fMRI, functional magnetic resonance imaging; SVM, support vector machine.

Next, we turned to the neuroimaging data to identify neural substrates of state-dependent changes in olfactory perceptual decision-making. As a first step, we used multivoxel pattern analysis to isolate brain regions in which neural patterns of food and nonfood odors could be reliably discriminated. To that end, we trained a support vector machine (SVM) classifier to distinguish patterns of fMRI activity evoked by pure food odor trials versus pure nonfood odor trials (**Fig 3A**). We implemented this analysis using a whole-brain searchlight approach and found significant (*p* < 0.05, whole-brain voxel-wise family-wise error [FWE] corrected) food versus nonfood odor decoding accuracy in bilateral inferior frontal and medial temporal lobes (left: x = −28, y = −6, z = −18 [Montreal Neurological Institute (MNI) coordinates], t_(29)_ = 6.81, *p*_*FWE*_ = 0.003; right: x = 20, y = −8, z = −18, t_(29)_ = 10.11, *p*_*FWE*_ < 0.001). These clusters included parts of piriform cortex and extended into amygdala, hippocampus, and parahippocampal gyrus (**Fig 3B, S1 Table**), and we refer to them as olfactory/limbic regions moving forward. In addition, we found significant food versus nonfood decoding accuracy in right mid-insula (x = 40, y = −2, z = 2, t_(29)_ = 7.85, *p*_*FWE*_ < 0.001; Fig 3C). Because these brain areas represent food versus nonfood odors, we selected them as regions of interest (ROIs) for subsequent analyses exploring satiety-based modulation of food versus nonfood odor encoding.

### Satiety reduces fMRI pattern discrimination of the meal-matched odor pair

**Fig 4:**
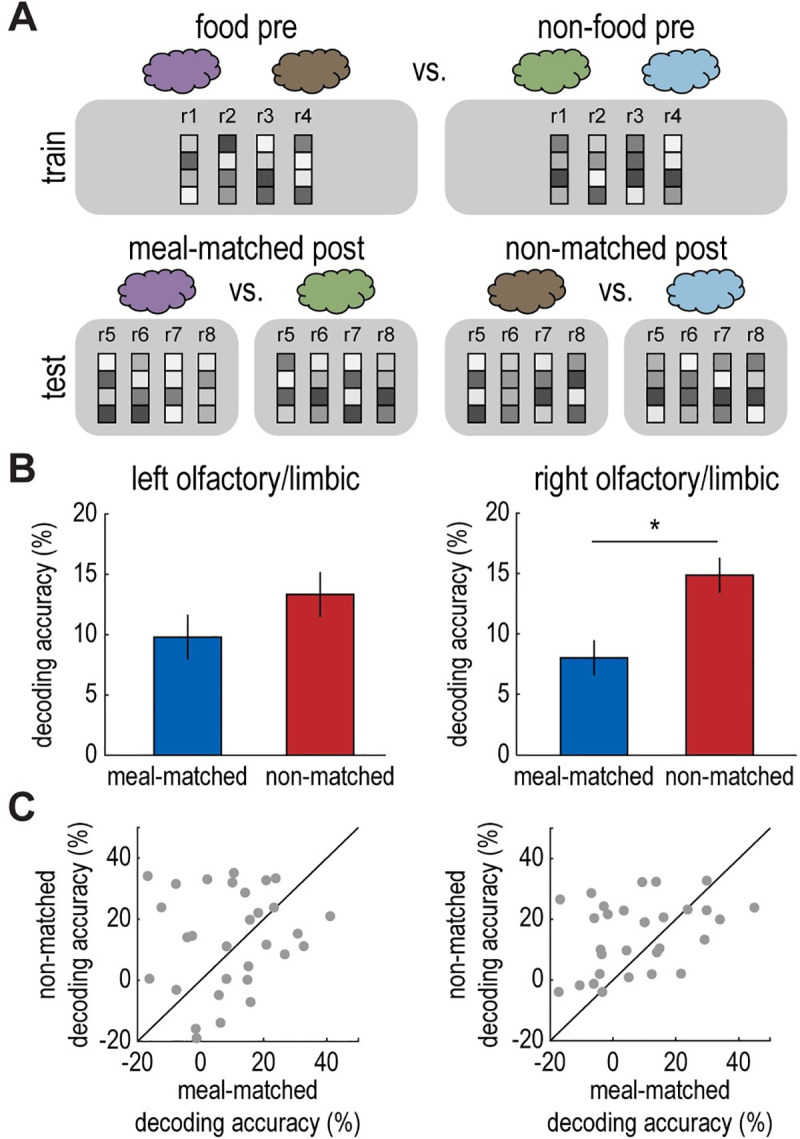
Satiety reduces fMRI pattern discrimination of the meal-matched odor pair. **(A)** Schematic of decoding analysis. An SVM classifier was trained to discriminate fMRI activity patterns evoked by pure food (average of cinnamon bun and pizza) vs. nonfood (average of cedar and pine) odors in the pre-meal session. The SVM classifier was tested on fMRI activity patterns evoked by pure odors in the post-meal session for meal-matched and non-matched conditions separately (cinnamon bun vs. cedar and pizza vs. pine). **(B)** In the right olfactory/limbic ROI (**Fig 3B**), food vs. nonfood decoding accuracy was significantly lower for the meal-matched odor pair compared to the non-matched odor pair in the sated state (t_(29)_ = 2.35, *p* = 0.03; right). This was not the case in the left olfactory/limbic ROI (t_(29)_ = 0.95, *p* = 0.35; left). **(C)** Individual participant data depicting post-meal decoding accuracy in the left and right olfactory/limbic ROIs for meal-matched vs. non-matched odor pairs. Error bars depict within-subject SEM for *n* = 30. Individual participant data summarized in these plots can be found in **S1 Data**. fMRI, functional magnetic resonance imaging; ROI, region of interest; SEM, standard error of the mean; SVM, support vector machine.

As a next step, we aimed to understand how meal consumption alters neural odor representations in the olfactory/limbic and insular regions identified by the previous analysis. Based on the behavioral data, we expected to observe a decrease in food versus nonfood odor discrimination for the meal-matched odor pair compared to the non-matched odor pair in the sated state. To test this prediction, we trained an SVM classifier to discriminate fMRI activity patterns evoked by pure food versus nonfood odors prior to the meal (**Fig 4A**). Then, we tested the classifier on fMRI activity patterns evoked by pure food versus nonfood odors after the meal in each of the ROIs, for meal-matched and non-matched odor pairs separately. Finally, we compared classifier accuracy across meal-matched and non-matched conditions (note that this ROI selection was based on the ability to discriminate food versus nonfood odors across both meal-matched and non-matched conditions and thus was not biased to find differences between meal-matched and non-matched odor pairs).

This analysis revealed that post-meal food versus nonfood odor decoding accuracy was indeed reduced for the meal-matched odor pair compared to the non-matched odor pair in the right olfactory/limbic ROI (t_(29)_ = 2.35, *p* = 0.03; **Fig 4B and 4C**). We did not identify a similar effect in the left olfactory/limbic ROI (t_(29)_ = 0.95, *p* = 0.35; **Fig 4B and 4C**) or the right insula ROI (t_(29)_ = 0, *p* = 1.00). Taken together, these findings demonstrate a meal-specific change in basic food versus nonfood odor discrimination in olfactory/limbic brain areas, which may underlie the meal-specific behavioral changes in perceptual decision-making.

### Satiety renders fMRI ensemble patterns of meal-matched odor mixtures less “food-like”

**Fig 5:**
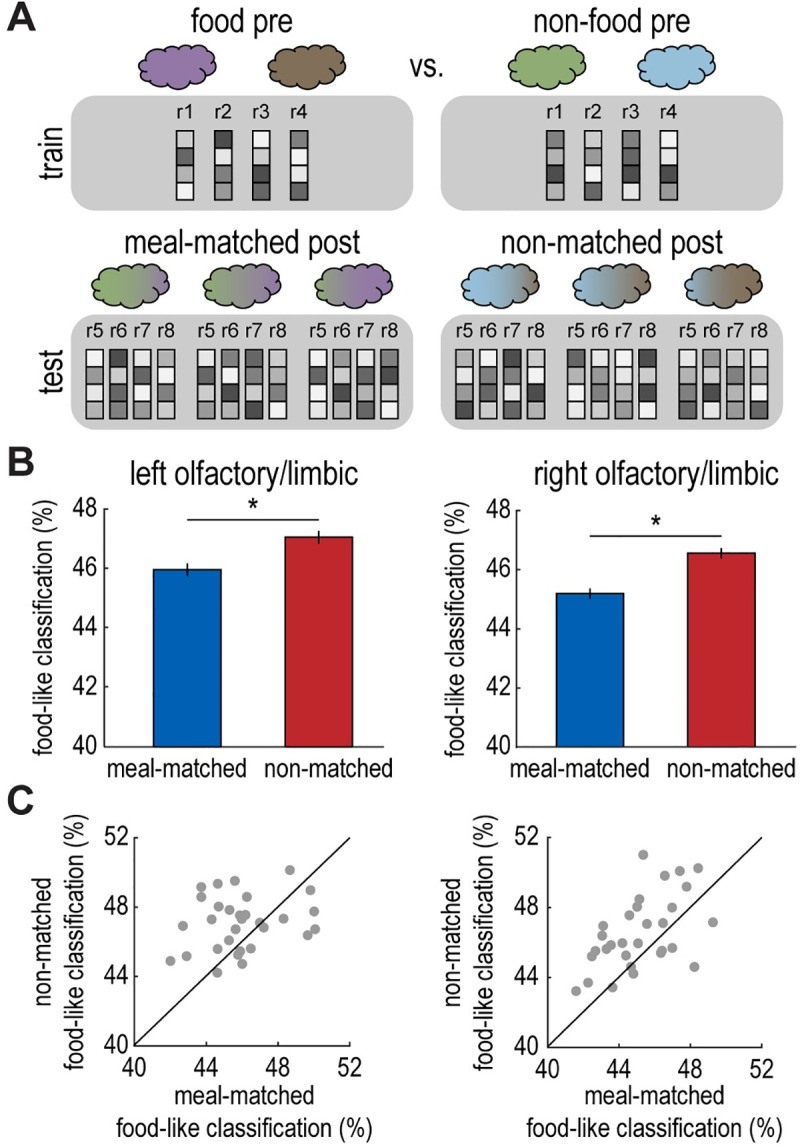
Satiety renders fMRI ensemble patterns of meal-matched odor mixtures less “food-like”. **(A)** Schematic of decoding analysis. An SVM classifier was trained to discriminate fMRI activity patterns evoked by food (average of cinnamon bun and pizza) vs. nonfood (average of cedar and pine) odors in the pre-meal session. The SVM classifier was tested on fMRI activity patterns evoked by each individual mixture (3 mixtures per odor pair), and then food-like values were averaged across mixtures for meal-matched and non-matched conditions separately (cinnamon bun/cedar mixtures and pizza/pine mixtures). **(B)** The SVM classifier identified fMRI patterns as food-like less often for meal-matched mixtures than non-matched mixtures in the left and right olfactory/limbic ROIs depicted in Fig 3B (left, t_(29)_ = 2.57, *p* = 0.02; right, t_(29)_ = 3.78, *p* < 0.001). **(C)** Individual participant data depicting food-like classification values in olfactory/limbic ROIs for meal-matched vs. non-matched odor mixtures. Error bars depict within-subject SEM for *n* = 30. Individual participant data summarized in these plots can be found in **S1 Data**. fMRI, functional magnetic resonance imaging; ROI, region of interest; SEM, standard error of the mean; SVM, support vector machine.

The previous findings indicate a state-dependent shift for neural representations of pure food and nonfood odors in olfactory/limbic brain areas. In a next step, we were interested in whether food consumption biases representations of the more ambiguous odor mixtures. Since sated participants were less likely to perceive the meal-matched food odor as dominant in a mixture, we predicted that fMRI ensemble patterns evoked by meal-matched odor mixtures would parallel these changes. Specifically, we hypothesized that meal-matched patterns would be less food-like compared to patterns evoked by non-matched odor mixtures post-meal. To test this prediction, we applied the SVM classifier that was trained on pure food versus nonfood fMRI activity patterns before the meal to patterns evoked by odor mixtures after the meal for meal-matched and non-matched odor pairs separately (**Fig 5A**). This way, we could determine whether neural responses to post-meal mixtures were more or less food-like based on their overlap with pure odor template patterns.

In line with our hypothesis, we found that the SVM classifier was less likely to identify post-meal odor mixture patterns as food-like for the meal-matched odor pair compared to the non-matched odor pair in left and right olfactory/limbic ROIs (left: t_(29)_ = 2.57, *p* = 0.02; right: t_(29)_ = 3.78, *p* < 0.001; **Figs 5B, 5C and S6**). However, we did not observe a significant difference between meal-matched and non-matched odor mixtures in the right insula ROI (t_(29)_ = 1.23, *p* = 0.23). These results demonstrate that olfactory/limbic brain regions represent meal-matched odor mixtures as less food-like in the sated state. These findings mirror our main behavioral result and suggest that sated participants’ perceptual bias could be driven by a shift toward nonfood-like neural representations in olfactory/limbic brain areas.

### Functional connectivity in response to food versus nonfood odors

**Fig 6:**
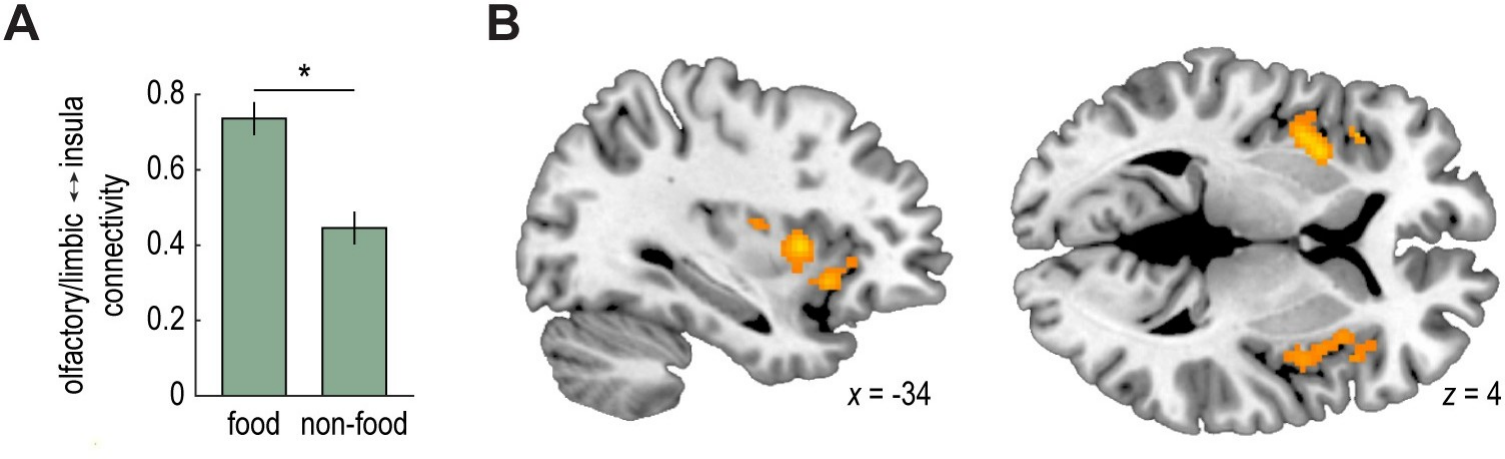
Functional connectivity in response to food versus nonfood odors. Enhanced functional connectivity between an olfactory/limbic seed region and insula during food vs. nonfood odor trials. This finding was based on the functionally defined ROI in right insula (t_(29)_ = 2.73, *p* = 0.01; A), and regional specificity was further confirmed by a voxel-wise test using anatomically defined ROIs in left and right insula (left insula: x = −34, y = 8, z = 4, t_(29)_ = 5.10, *p*_*FWE-*SVC_ = 0.01; right insula: x = 40, y = 4, z = −6, t_(29)_ = 4.36, *p*_*FWE-*SVC_ = 0.04; B). The group-level t-map in (B) is thresholded at *p*_*unc*_ < 0.005, masked with an anatomical insula ROI (AAL atlas), and overlaid on a canonical structural image. Individual participant data summarized in these plots can be found in **S1 Data**, and the whole-brain statistical map can be viewed at neurovault.org/collections/EWYJXOKG/images/510269/. AAL, automated anatomical labeling; ROI, region of interest.

The results above show that fMRI ensemble patterns in olfactory/limbic brain areas are modulated by motivational state. In a next step, we tested the effect of food intake on functional interactions between this region and other areas of the network, specifically the insula. To that end, we conducted a generalized psychophysiological interaction (PPI) analysis [52] where the seed region was the right olfactory/limbic cluster, and the psychological factors were odor type (pure food odor trials versus pure nonfood odor trials) and session (pre-meal versus post-meal). Note that odor trials were pooled across meal-matched and non-matched odor pairs for this initial PPI analysis. We found that the connectivity between the right olfactory/limbic seed and insular cortex (both defined based on the results depicted in **Fig 3**) was significantly stronger for pure food versus pure nonfood odor trials across both sessions (t_(29)_ = 2.73, *p* = 0.01; **Fig 6A**). To determine the regional specificity of this finding, we tested voxel-wise PPI estimates in anatomical ROIs of left and right insula (automated anatomical labeling [AAL] atlas). This revealed significant (*p* < 0.05, FWE small volume corrected) clusters of connectivity (pure food odor > pure nonfood odor) in bilateral mid/anterior insula (left insula: x = −34, y = 8, z = 4, t_(29)_ = 5.10, *p*_*FWE-*SVC_ = 0.01; right insula: x = 40, y = 4, z = −6, t_(29)_ = 4.36, *p*_*FWE-*SVC_ = 0.04; **Fig 6B**).

In a subsequent PPI analysis, we tested whether the change in food versus nonfood odor connectivity between olfactory/limbic and insular ROIs was different from pre- to post-meal for the specific meal-matched and non-matched odor pairs. Contrary to our prediction, we did not observe a significant 3-way interaction (food odor > nonfood odor by pre-meal > post-meal by meal-matched > non-matched; t_(29)_ = 0.03, *p* = 0.98). However, we found a significant 2-way interaction (food odor > nonfood odor by pre-meal > post-meal), such that the difference in food versus nonfood odor connectivity was reduced in the sated state compared to the fasted state (pre-meal: t_(29)_ = 3.33, *p* = 0.002; post-meal: t_(29)_ = 0.26, *p* = 0.79; odor by session interaction: t_(29)_ = 2.62, *p* = 0.01; **S7 Fig**). Although this finding tentatively suggests that the connectivity between olfactory/limbic and insular brain regions is modulated by satiety state, irrespective of odor identity, it should be interpreted with caution, as it could also be driven by the passage of time or task repetition.

## Discussion

Our results demonstrate that motivational state modulates olfactory perceptual decision-making and odor-evoked brain activity in humans. Behaviorally, participants’ perceptual choices were biased away from meal-matched food odors in the sated state, while their choices regarding an alternative food odor did not change. The ability of the olfactory system to selectively bias perceptual decision-making for specific food odors based on the content of a recent meal could serve as an adaptive strategy to optimize food search, ultimately promoting variety in nutrient intake.

Binary odor mixtures have been widely used in previous rodent work to study olfactory perceptual decision-making [31,32,53–56]. These studies have yielded important insights regarding how factors such as speed, accuracy, and confidence modulate perceptual choices. In contrast, human research on olfactory perceptual decision-making is more limited. One prior study found fMRI evidence that orbitofrontal cortex accumulates information across serial sniffs, while piriform cortex provides an ongoing sensory report of odor input [33], and a different study implicated medial prefrontal cortex in subjective judgments of odor value [57]. However, while satiety has been shown to influence configural and elemental aspects of olfactory processing [18], whether and how satiety biases perceptual decision-making have not been tested.

Our findings address this question. At the neural level, we found that odor-evoked activity in olfactory/limbic brain regions was altered by food intake in an odor-specific manner. Specifically, in the sated state, multivoxel patterns of food and nonfood odors were less discriminable for the meal-matched odor pair than the non-matched odor pair. Furthermore, neural patterns of meal-matched odor mixtures were less food-like compared to their non-matched counterparts. These results extend prior rodent work investigating the effects of satiety on odor sensitivity and early olfactory signaling. In one such study, the scent of food pellets evoked stronger neural responses in olfactory bulb in fasted compared to sated rats [39]. Along similar lines, satiety has been shown to decrease neural discrimination of odors in the mouse olfactory bulb [41]. However, these previous animal studies did not examine the effect of satiety on the encoding of meal-matched versus non-matched food odors, leaving it open whether satiety modulates olfactory processing in an odor-specific manner. The results from our pattern-based analyses address this gap, demonstrating that satiety modulates neural representations of specific odors during perceptual decision-making.

Notably, due to technical limitations of fMRI, it is not possible to measure neural activity in olfactory bulb. It is therefore unclear whether the odor-specific changes observed in olfactory/limbic brain regions have even earlier origins. In addition, since our experiment was limited to 2 odor pairs, we cannot assess the extent to which these meal-specific effects generalize to related food odors. For instance, does filling up on pizza alter perceptual choices and neural responses for pizza odor in isolation or for the scent of savory food odors more broadly? Future work could address this question by exploring whether satiety modulates olfactory perceptual decision-making in an identity-specific or category-specific manner.

Interestingly, we also found that the functional connectivity between the olfactory/limbic region and insula was enhanced for food odors when compared to nonfood odors. This connectivity difference was larger in the pre-meal session compared to the post-meal session, but this effect was not specific to the meal-matched odor. Thus, these results could be driven by time or task repetition, rather than satiety. As such, they should be interpreted with caution. However, they may suggest a more general role for insula in conveying the biological relevance of olfactory food signals in accordance with motivational states. Indeed, recent rodent work has demonstrated that insula neurons show increased firing in response to food-predictive cues in fasted, but not sated, animals [58]. Similarly, previous human research has shown that odor-evoked connectivity between piriform cortex and insula mediates changes in food selection in the sleep-deprived state [42].

More generally, we found that fMRI ensemble patterns associated with food and nonfood odors were discriminable in mid-insular cortex. These pattern differences may have been driven by olfactory or trigeminal qualities of the complex odor stimuli. In either case, previous studies in humans have also demonstrated that this part of the insula encodes taste quality [59–62] and preferentially responds to food-based visual stimuli [43,63,64]. Moreover, recent rodent work has shown that taste quality is represented via distributed codes in mouse insula [65,66]. Our findings complement these previous studies and further support the idea that insula integrates food-related signals across sensory modalities.

While our results suggest that hunger and satiety bias olfactory perceptual decision-making, the reasons for this shift warrant further exploration. Our imaging findings suggest a role for relatively early processing in piriform cortex and amygdala, but food intake could also modulate perceptual choices via top-down attentional mechanisms or value-based mechanisms affecting the desirability of specific food odors. Paradigms involving more complex behavioral tasks are needed to disentangle these possibilities, both to probe perceptual decisions in more naturalistic scenarios, and to replicate and extend the current findings.

In summary, our behavioral results demonstrate that olfactory perceptual choices can be flexibly altered to accommodate the current motivational state. Moreover, our neural findings suggest a potential mechanism for this behavioral shift via odor-specific modulation of brain activity in olfactory/limbic areas.

## Materials and methods

### Ethics statement

The study protocol was approved by the Northwestern University Institutional Review Board (STU00098371), and the experiments were conducted according to the principles expressed in the Declaration of Helsinki. All subjects gave written informed consent to participate.

### Participants

A total of 32 healthy human participants (19 females, mean age: 23.03 years, age range: 18 to 30 years) took part in the study. Participants were right-handed and did not have MRI contraindications (metal implants, claustrophobia, etc.). Exclusionary criteria included history of psychiatric or neurological conditions, use of psychotropic medications, habitual smoking, smell or taste dysfunction, severe food or nonfood allergies, history of eating disorders, and ongoing dieting or fasting. Critically, participants confirmed their willingness to eat either of the experimental meals (i.e., cinnamon buns or pizza) prior to the study, although they were not made aware of their group assignment until the meal phase of the experiment. One participant was excluded from analysis for misunderstanding perceptual decision-making task instructions, and another was excluded for missing a large number of responses during the task (more than 15% of trials). This left 30 total participants for analysis.

### Olfactory stimuli

The stimulus set included 2 familiar food odors (i.e., cinnamon bun and pizza) and 2 familiar nonfood odors (i.e., cedar and pine). For the perceptual decision-making task, food and nonfood odors were combined to make binary odor mixtures. The goal was to identify odors that complemented each other well, while remaining distinct enough that participants could detect the individual components in a mixture. We conducted extensive pilot testing to confirm this was the case for the 2 odor pairs that were chosen (i.e., cinnamon bun and cedar and pizza; pine). Notably, odors were complex stimuli, some of which contained trigeminal components. Thus, both olfactory and trigeminal qualities likely contributed to the behavioral and neural findings.

Pure odors and odor mixtures were presented to participants via a computer-controlled olfactometer at a steady flow rate of 3.2 L/min, as in our previous studies [16,42,67]. The olfactometer included 2 mass flow controllers (Alicat Scientific, Tucscon, Arizona, USA) that operated independently, so pure odors could be diluted with odorless air, and food and nonfood food odors could be combined to form custom mixtures.

### Experimental design

Prior to the main experiment, participants came to the lab for a pre-study visit, where they were fitted for custom Styrofoam head cases (Caseforge, Berkeley, CA, USA) and familiarized with study procedures. At a later date, participants returned to the lab for the main experiment, which took approximately 3 hours to complete. Participants were instructed to refrain from consuming food or beverages (except for water) for at least 6 hours leading up to the main experiment so they would be hungry when they arrived. During the main experiment, participants completed 2 olfactory task sessions while undergoing MRI scanning. These consisted of an odor rating task followed by a perceptual decision-making task. The 2 sessions were separated by an experimental meal, and they were identical except that the order of trials was pseudorandomized independently for each session.

#### Odor rating task

During the odor rating task, participants rated the 4 pure odors (i.e., cinnamon bun, cedar, pizza, and pine) in terms of pleasantness (from “extremely unpleasant” to “extremely pleasant”), intensity (from “extremely weak” to “extremely strong”), and food-likeness (from “not at all food-like” to “extremely food-like”). For the food-likeness metric, participants were instructed to make their ratings based on the food-like quality of the odors more generally and not in relation to any specific food. On each trial, an odor label appeared above a countdown (2 seconds). Participants were cued to sniff upon odor presentation, and then they used a mouse to navigate a sliding scale and submit their rating.

There were 3 rating trials per odor per condition (for a total of 36 trials), and trial order was pseudorandomized to ensure that the same odor would not be presented for more than 2 consecutive trials. Participants completed the odor rating task during structural MRI acquisition.

#### Olfactory perceptual decision-making task

During the perceptual decision-making task, participants were presented with pure odors, odor mixtures, and odorless air. Odor mixtures contained food and nonfood components (i.e., either cinnamon bun and cedar or pizza and pine) in variable concentrations. On each trial, 2 odor labels appeared on either side of a countdown (2 seconds), so participants were aware of the trial type prior to odor exposure. The label positions were counterbalanced across trials to de-correlate perceptual choices from visual and motor signals. Participants were cued to sniff upon odor delivery (2 seconds), and then they made a perceptual decision regarding which component was dominant in the mixture. Participants used the left and right mouse buttons to indicate their choice as quickly and accurately as possible. They were given 2 seconds to respond, and missed responses prompted a “TOO SLOW” message. After the decision phase, there was a variable delay (3 to 7 seconds) before the next trial. Thus, the time between the onset of 2 consecutive odor presentations was always at least 9 (and up to 13) seconds to prevent habituation.

In each session (i.e., pre- or post-meal), each unique odor was presented 16 times (for a total of 160 odor trials per session). Trial order was pseudorandomized to ensure that the same odor pair would not be presented for more than 5 consecutive trials, and the same specific odor would not be presented for more than 2 consecutive trials. In addition, there were 32 odorless air trials, and participants were instructed to press either mouse button upon receiving odorless air. Participants completed the perceptual decision-making task during fMRI acquisition, and each session was divided into 4 equal runs of approximately 8.5 minutes each.

#### Experimental meal

After the first session of olfactory tasks, participants were given an experimental meal. The meal consisted of cinnamon buns or cheese pizza depending on group assignment. The food items were selected to match the food odors as closely as possible, and water was provided. Participants were instructed to eat as much as they could until they were very full, and then they were left alone for 15 minutes. After the meal, participants were escorted back to the MRI scanner room for the second session of olfactory tasks. Notably, participants consumed an average of 833.37 calories ± 49.20 standard error of the mean (SEM) during the meal phase (cinnamon buns: 805.82 calories ± 81.33; pizza: 860.91 calories ± 59.49).

#### Appetite rating task

Appetite ratings were collected at 4 time points during the main experiment, once at the beginning and once at the end of each of the 2 sessions. Participants answered 3 appetite-related questions at each time point (“How hungry do you feel?,” “How full do you feel?,” and “How much food do you feel you can eat right now?”). Participants used a mouse to navigate a sliding scale and submit their ratings.

#### Post-experiment questionnaire

After the second session, participants completed a brief survey where they answered 9 experiment-related questions. Participants used a mouse to navigate a sliding scale and submit their ratings. The first 4 questions assessed the authenticity of each of the 4 odor stimuli (e.g., “How much did the cinnamon bun odor smell like cinnamon bun?,” from not at all [0] to moderately [50] to very much [100]). Ratings indicated that participants generally found the odor stimuli to be fairly authentic (cinnamon bun, mean = 84.53 ± 2.98 SEM; cedar, mean = 68.68 ± 4.01 SEM; pizza, mean = 46.19 ± 4.86 SEM; pine, mean = 55.46 ± 4.38 SEM).

The next 2 questions pertained to the meal phase (“How well did the meal match the odor?,” from not at all [0] to moderately [50] to very well [100]; “How much did you enjoy eating the food during the study?,” from not at all [0] to moderately [50] to very much [100]). Ratings suggested that the experimental meal and paired odor were moderately well matched (mean = 52.56 ± 4.65 SEM) and that participants enjoyed the meal (mean = 70.08 ± 3.84 SEM). For the remaining questions, participants rated their sense of smell and engagement over the course of the experiment.

### MRI acquisition and preprocessing

MRI data were collected using a 3T Siemens PRISMA system (Munich, Germany) and a 64-channel head coil. During MRI scanning, custom Styrofoam head cases (Caseforge) were used to minimize head motion. A T1-weighted structural scan was acquired for each participant at the start of each MRI session (repetition time [TR], 2,170 ms; echo time [TE], 1.69 ms; flip angle, 7°; 1 mm isotropic voxels; no gap; number of slices, 256; field of view, 176 mm × 256 mm × 256 mm). The 2 T1-weighted images were co-registered and averaged, and the resulting mean image was used for normalization to MNI space. Functional echo-planar images (EPIs, 262 per run) were acquired with the following parameters: TR, 2,000 ms; TE, 22 ms; flip angle, 80°; field of view, 208 mm × 192 mm × 116 mm; in-plane resolution, 2 mm × 2 mm; slice thickness, 2 mm, no gap; number of slices, 58; interleaved slice acquisition order; multiband factor, 2. EPIs were acquired at a tilted acquisition angle (approximately 20° from axial) to minimize signal dropout in olfactory areas [68,69]. Depending on the participant, functional EPIs covered the whole brain, with the occasional exception of the most dorsal part of the parietal lobe. A total of 10 whole-brain EPIs were also acquired to aid with co-registration between the anatomical and functional images. These images were acquired using the same acquisition parameters, with the following exceptions: TR, 3,150 ms; number of slices, 96; field of view, 208 mm × 192 mm × 192 mm.

MRI data were preprocessed and analyzed using SPM12 software (Wellcome Centre for Human Neuroimaging, London, UK) (www.fil.ion.ucl.ac.uk/spm/). To account for head motion, functional EPIs were realigned to the first image. In addition, whole-brain EPIs were realigned and averaged. The average whole-brain EPI was co-registered to the average T1-weighted image, the average functional EPI was co-registered to the average whole-brain EPI, and these transformations were applied to the EPI time series data. Spatial normalization was accomplished by (1) normalizing the average T1-weighted image to the MNI template (using the 6-tissue probability map from SPM12); and (2) applying the resulting deformation fields to the EPIs. Before pattern-based and functional connectivity analyses, normalized images were smoothed using 2 mm^3^ and 6 mm^3^ Gaussian kernels, respectively. The maps resulting from searchlight analyses were smoothed using a 6 mm^3^ Gaussian kernel prior to statistical group-level analysis.

### fMRI multivoxel pattern analysis

To identify brain areas in which patterns of fMRI activity could be discriminated between food and nonfood odors, we used a searchlight-based pattern analysis. First, a general linear model (GLM) was constructed for each participant. The odor trials (i.e., onsets of the sniff cues) were modeled as events, and trials were collapsed across the 2 odor pairs, such that food odor trials (i.e., cinnamon bun and pizza) were modeled together, and nonfood odor trials (i.e., cedar and pine) were modeled together. The model contained additional regressors representing the various odor mixture trials (also collapsed across odors pairs), air trials, and left and right button presses (also modeled as events). Nuisance regressors included (1) the 6 standard motion parameters from realignment and their squares, derivatives, and squared derivatives (24 regressors total); (2) 4 additional regressors to account for head motion within scans (signal difference between even and odd slices, variance across slices for each volume, derivatives of both measures, and extra regressors to account for individual volumes with excessive within-scan head motion as needed); (3) a respiration regressor derived from the breathing trace (acquired using a breathing belt [BIOPAC Systems, Goleta, CA, USA]) that was postprocessed and down-sampled to match the scanner TR; and (4) run-wise dummy regressors. Within each GLM, odor and air trials were modeled separately for each run, while button presses and nuisance regressors were concatenated across runs to avoid overfitting. After GLM estimation, t-maps corresponding to food and nonfood conditions (2 conditions × 8 runs) were computed for each participant for pattern analysis.

Pattern analysis was implemented throughout the whole brain using a searchlight-based approach [70–72], where the search sphere encompassed an approximately 4-voxel radius. T-map patterns were extracted at each search sphere, and the mean across conditions was subtracted within each run. An SVM classifier (LIBSVM) was trained and tested on the resulting patterns using a leave-one-run-out cross-validation approach. More specifically, the classifier was trained to discriminate food and nonfood odor patterns based on 7 runs and then tested on data from the left-out run. Decoding accuracy values were averaged across all 8 iterations, and the mean accuracy at each search sphere was assigned to the center voxel to construct a decoding accuracy map. Individual accuracy maps were smoothed and subjected to group-level *t* tests. ROIs in left and right olfactory/limbic brain regions, and in right mid-insula, were defined for subsequent analyses based on the resulting group-level statistical map (thresholded at *p*_*FWE*_ < 0.05).

To measure state-dependent changes in food versus nonfood odor pattern discrimination, similar steps were repeated as for the pattern analysis described above, with a few exceptions. First, the SVM classifier was trained exclusively on food versus nonfood odor patterns from the pre-meal session (2 conditions × 4 runs). Second, the classifier was tested on post-meal patterns extracted from a different GLM, which was identical to the original GLM, except that odor trials were modeled separately for the 2 odor pairs (4 conditions × 4 runs). Thus, the classifier could determine food versus nonfood decoding accuracy in the sated state for meal-matched and non-matched odor pairs separately. Decoding accuracy values for meal-matched and non-matched conditions were extracted from the previously defined ROIs in left and right olfactory/limbic brain regions, and in right insula, on a participant-by-participant basis and averaged across voxels prior to comparison across conditions.

To measure state-dependent changes in the “food-like” quality of fMRI patterns evoked by odor mixtures, the previous analysis was repeated with one exception. In this case, the SVM classifier was tested on activity patterns evoked by post-meal mixtures from both meal-matched and non-matched odor pairs (6 conditions × 4 runs). Food-like classification values were averaged across the 3 mixtures for meal-matched and non-matched odor pairs separately, and the average value was used to construct food-like maps (one meal-matched map and one non-matched map). In a complementary analysis, food-like classification values were kept separate for each of the ten odors (5 odors [2 pure and 3 mixtures] × 2 conditions) instead of averaging across the 3 odor mixtures, which resulted in 10 food-like maps (5 meal-matched maps and 5 non-meal-matched maps). In either case, food-like classification values were extracted from meal-matched and non-matched maps based on the previously defined ROIs in left and right olfactory/limbic brain regions, and in right insula, on a participant-by-participant basis and averaged across voxels prior to comparison across conditions.

### fMRI connectivity analysis

The gPPI toolbox [52] was used to measure the functional connectivity between olfactory/limbic and insular ROIs. The physiological factor was fMRI activity in a seed region in the right olfactory/limbic ROI, which was defined based on the food versus nonfood odor decoding analysis described previously. The psychological factor was pure food and nonfood odor trials (collapsed across the odor pairs), modeled separately for pre- and post-meal sessions. This resulted in a voxel-wise map of food versus nonfood odor functional connectivity estimates with the seed region. Connectivity estimates for food and nonfood conditions were extracted from the previously defined ROI in right insula on a participant-by-participant basis and averaged prior to comparison across conditions. In a complementary analysis to confirm regional specificity, the same connectivity maps were analyzed on a voxel-wise level within 2 anatomically defined ROIs of bilateral insula (AAL atlas). Similar steps were repeated to measure pre- to post-meal connectivity changes for meal-matched and non-matched conditions separately, except that pure food and nonfood odors corresponding to each of the 2 odor pairs were considered separately for the psychological factor.

### Statistical analysis

Paired *t* tests were utilized to compare behavioral data across conditions, and a 2-sample *t* test was used to test for sex differences. All behavioral tests were 2-tailed unless otherwise noted. To identify brain areas with above-chance decoding accuracy for discriminating food versus nonfood odors, 1-tailed voxel-wise *t* tests were carried out in SPM12. Results were whole-brain FWE corrected at the voxel level, and ROIs were defined using a significance threshold of *p*_*FWE*_ < 0.05. For analyses within these functionally defined ROIs, values were extracted from decoding or connectivity maps and compared across conditions using 2-tailed paired *t* tests. For the voxel-wise connectivity analysis, results from 1-tailed *t* tests were small volume corrected based on separate ROIs in left and right insula defined using the AAL atlas. All *p*-values from group-level fMRI maps were defined based on the peak voxel.

## Supporting information

S1 Fig Appetite ratings. **(A–C)** Participants’ appetites decreased significantly from before to after the experimental meal. Specifically, participants reported that they felt less hungry (t_(29)_ = 19.31, *p* < 0.001; A), more full (t_(29)_ = 16.19, *p* < 0.001; B), and able to eat less food (t_(29)_ = 15.09, *p* < 0.001; C). Error bars depict within-subject SEM for *n* = 30. Individual participant data summarized in these plots can be found in **S1 Data**. SEM, standard error of the mean.
(TIF)

S2 Fig Odor ratings. **(A)** Participants rated food odors as significantly less pleasant in the sated state than in the fasted state (t_(29)_ = 8.19, *p* < 0.001), while there was a trend in the opposite direction for nonfood odors (t_(29)_ = 2.04, *p* = 0.05). Pleasantness ratings declined significantly from before to after the meal for both meal-matched (t_(29)_ = 6.43, *p* < 0.001) and non-matched (t_(29)_ = 5.15, *p* < 0.001) odors. **(B)** Participants rated food odors as significantly more intense in the sated stated than in the fasted state (t_(29)_ = 2.39, *p* = 0.02), whereas there was no change in intensity ratings for the nonfood odors (t_(29)_ = 0.56, *p* = 0.58). Intensity ratings increased significantly from pre- to post-meal for the non-matched odor (t_(29)_ = 2.17, *p* = 0.04), but not for the meal-matched odor (t_(29)_ = 1.90, *p* = 0.07). **(C)** Participants rated food odors as significantly less food-like in the sated state than in the fasted state (t_(29)_ = 2.34, *p* = 0.03), while there was no change in food-like ratings for the nonfood odor (t_(29)_ = 0.93, *p* = 0.36). Food-like ratings decreased significantly from pre- to post-meal for the meal-matched odor (t_(29)_ = 2.36, *p* = 0.03), but not for the non-matched odor (t_(29)_ = 1.80, *p* = 0.08). Error bars depict within-subject SEM for *n* = 30. Individual participant data summarized in these plots can be found in **S1 Data**. SEM, standard error of the mean.
(TIF)

S3 Fig Individual participant data depicting average perceptual choices across mixtures and fitted sigmoidal choice curves. Participants 1–15. Individual participant data shown in these plots can be found in **S1 Data**.
(TIF)

S4 Fig Individual participant data depicting average perceptual choices across mixtures and fitted sigmoidal choice curves. Participants 16–30. Individual participant data shown in these plots can be found in **S1 Data**.
(TIF)

S5 Fig Satiety does not influence olfactory acuity. **(A, B)** Individual participant data depicting the slope of the sigmoidal function pre- and post-meal for meal-matched (A) and non-matched (B) odor pairs. **(C)** There was no significant change in the sigmoidal slope from before to after the meal for the meal-matched odor pair (t_(29)_ = 1.46, *p* = 0.15) or the non-matched odor pair (t_(29)_ = 0.42, *p* = 0.68). Error bars depict within-subject SEM for *n* = 30. Individual participant data summarized in these plots can be found in **S1 Data**. SEM, standard error of the mean.
(TIF)

S6 Fig “Food-likeness” of fMRI ensemble patterns in the sated state across odor mixtures. The SVM classifier identified fMRI patterns as food-like less often for meal-matched mixtures than non-matched mixtures in left and right olfactory/limbic ROIs (**Fig 5**). When considering food-like classification values for each odor mixture separately in these ROIs, differences seem to be driven largely by the 2 mixtures closer to the nonfood side of the spectrum. Note that the large difference in classification is expected for the pure odor endpoints given that ROIs were defined based on ability to discriminate pure food vs. nonfood odors. Individual participant data summarized in these plots can be found in **S1 Data**. fMRI, functional magnetic resonance imaging; ROI, region of interest; SVM, support vector machine.
(TIF)

S7 Fig Functional connectivity in response to food versus nonfood odors for pre- versus post-meal sessions. Connectivity differences between food and nonfood odor trials were significant pre-meal (t_(29)_ = 3.33, *p* = 0.002), but not post-meal (t_(29)_ = 0.26, *p* = 0.79; odor by session interaction, t_(29)_ = 2.62, *p* = 0.01). This interaction was driven by an increase in nonfood odor connectivity from pre- to post-meal, while connectivity in response to food odors did not change. Error bars depict within-subject SEM for *n* = 30. Individual participant data summarized in these plots can be found in **S1 Data**. SEM, standard error of the mean.
(TIF)

S1 Table Brain regions that discriminate fMRI ensemble patterns of food and nonfood odors. The group-level t-map was thresholded at *p*_*FWE*_ < 0.05 with a minimum cluster size of 15 voxels. Individual participant data summarized in these plots can be found in **S1 Data**. fMRI, functional magnetic resonance imaging.
(DOCX)

S1 Data Excel spreadsheet containing individual participant data for Figs 1D, 1E, 2A–2D, 4B, 4C, 5B, 5C, and 6A and S1A–S1C, S2A–S2C, S3, S4, S5A–S5C, S6, and S7 Figs. (XLSX)

## Abbreviations

AAL: automated anatomical labeling
EPI: echo-planar image
fMRI: functional magnetic resonance imaging
FWE: family-wise error
GLM: general linear model
ITI: intertrial interval
MNI: Montreal Neurological Institute
PPI: psychophysiological interaction
ROI: region of interest
SEM: standard error of the mean
SVM: support vector machine
TE: echo time
TR: repetition time
