# Supplementary figures and images for: Olfactory perceptual decision-making is biased by motivational state
Source: PLoS Biol. 2021 Aug 26;19(8):e3001374. doi: 10.1371/journal.pbio.3001374 (PMC8389475; doi:10.1371/journal.pbio.3001374)

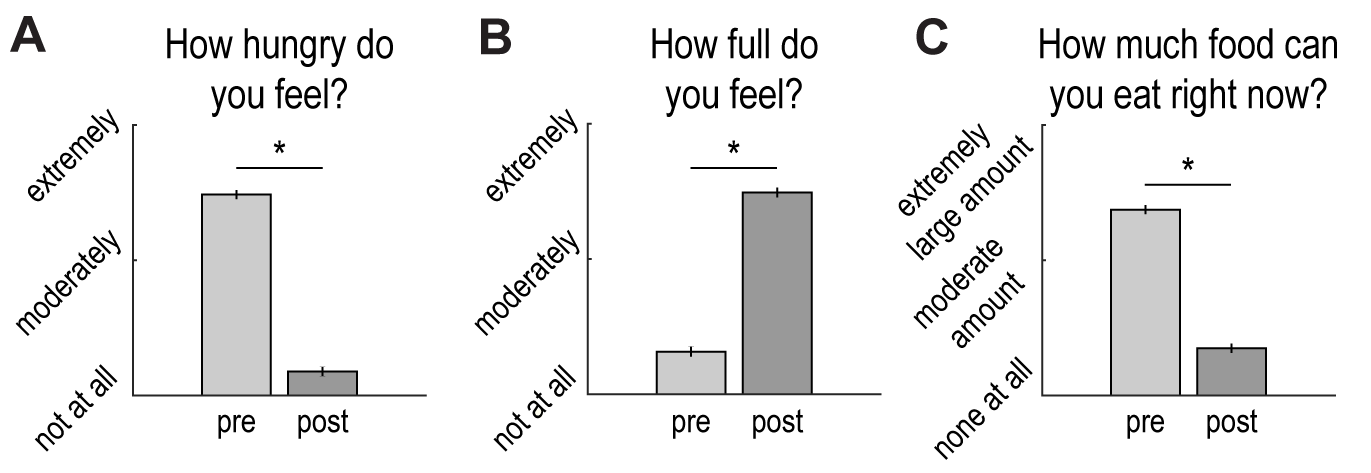

Supplement: S1 Fig — (A–C) Participants’ appetites decreased significantly from before to after the experimental meal. Specifically, participants reported that they felt less hungry (t(29) = 19.31, p < 0.001; A), more full (t(29) = 16.19, p < 0.001; B), and able to eat less food (t(29) = 15.09, p < 0.001; C). Error bars depict within-subject SEM for n = 30. Individual participant data summarized in these plots can be found in S1 Data. SEM, standard error of the mean. (TIF) [file pbio.3001374.s001.tif]

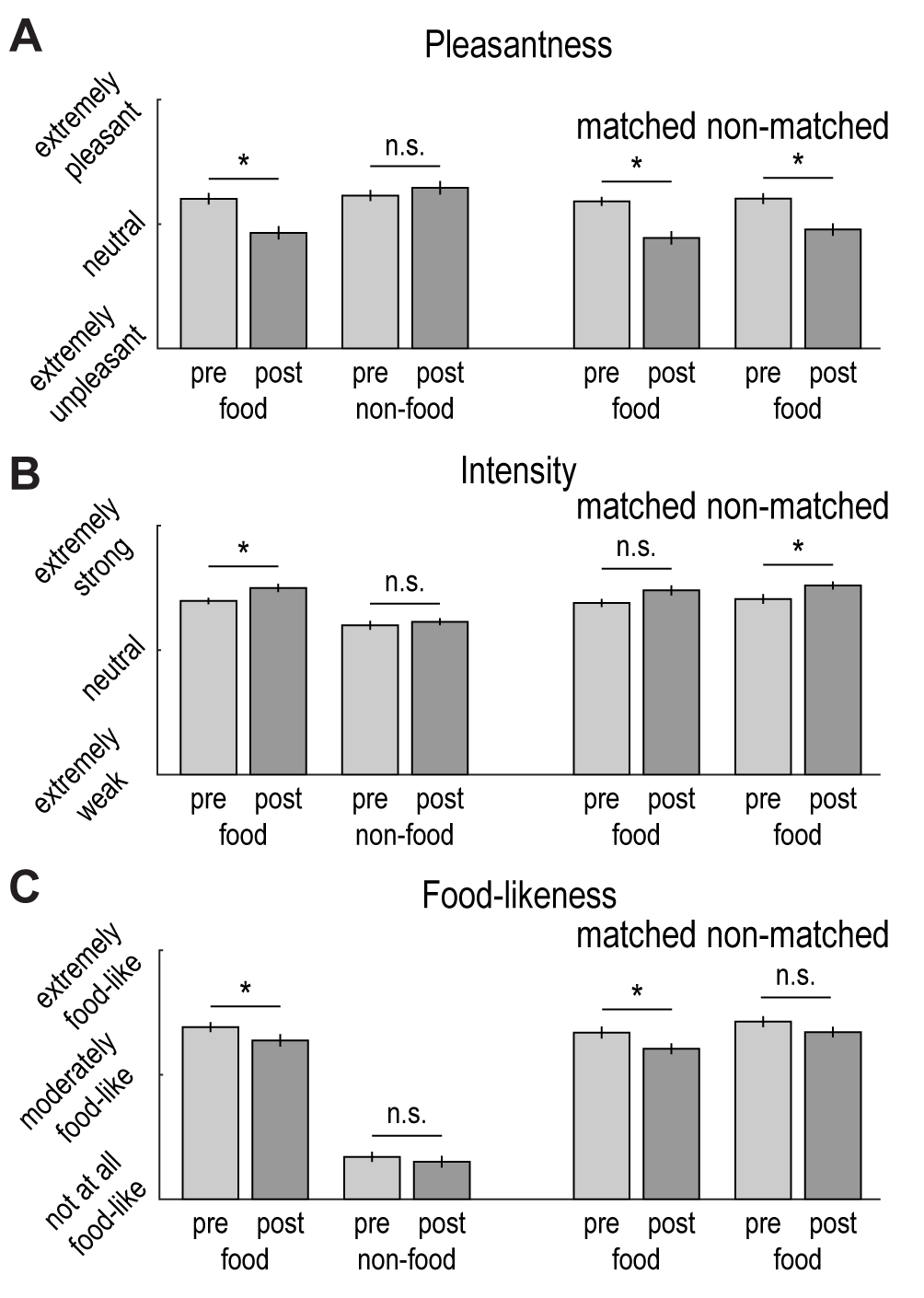

Supplement: S2 Fig — (A) Participants rated food odors as significantly less pleasant in the sated state than in the fasted state (t(29) = 8.19, p < 0.001), while there was a trend in the opposite direction for nonfood odors (t(29) = 2.04, p = 0.05). Pleasantness ratings declined significantly from before to after the meal for both meal-matched (t(29) = 6.43, p < 0.001) and non-matched (t(29) = 5.15, p < 0.001) odors. (B) Participants rated food odors as significantly more intense in the sated stated than in the fasted state (t(29) = 2.39, p = 0.02), whereas there was no change in intensity ratings for the nonfood odors (t(29) = 0.56, p = 0.58). Intensity ratings increased significantly from pre- to post-meal for the non-matched odor (t(29) = 2.17, p = 0.04), but not for the meal-matched odor (t(29) = 1.90, p = 0.07). (C) Participants rated food odors as significantly less food-like in the sated state than in the fasted state (t(29) = 2.34, p = 0.03), while there was no change in food-like ratings for the nonfood odor (t(29) = 0.93, p = 0.36). Food-like ratings decreased significantly from pre- to post-meal for the meal-matched odor (t(29) = 2.36, p = 0.03), but not for the non-matched odor (t(29) = 1.80, p = 0.08). Error bars depict within-subject SEM for n = 30. Individual participant data summarized in these plots can be found in S1 Data. SEM, standard error of the mean. (TIF) [file pbio.3001374.s002.tif]

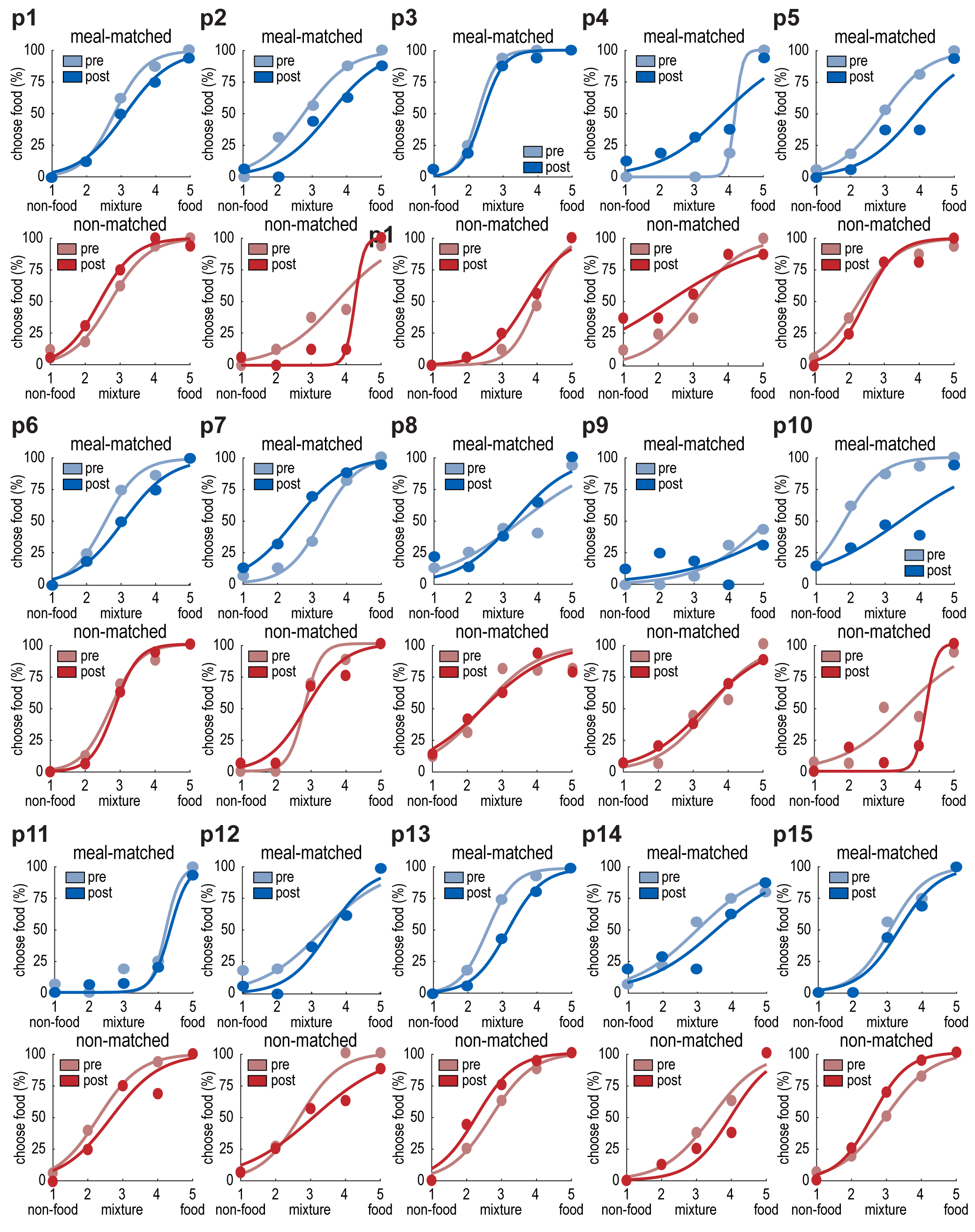

Supplement: S3 Fig — Participants 1–15. Individual participant data shown in these plots can be found in S1 Data. (TIF) [file pbio.3001374.s003.tif]

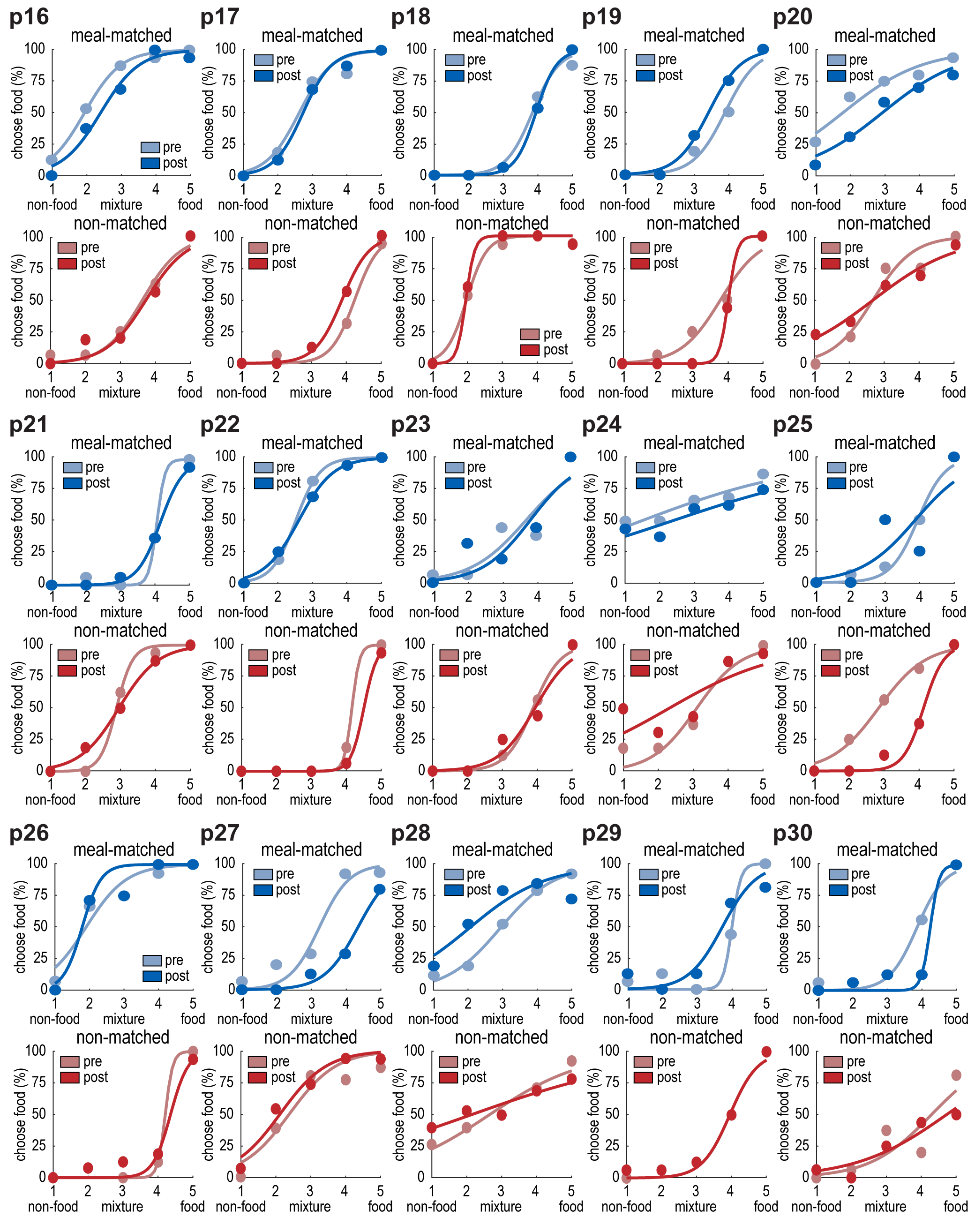

Supplement: S4 Fig — Participants 16–30. Individual participant data shown in these plots can be found in S1 Data. (TIF) [file pbio.3001374.s004.tif]

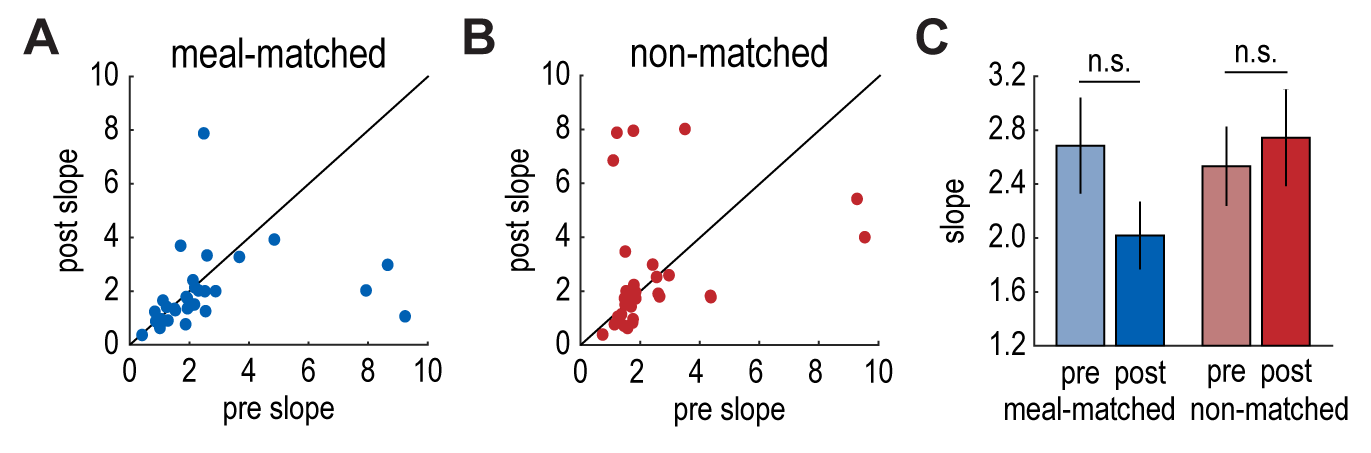

Supplement: S5 Fig — (A, B) Individual participant data depicting the slope of the sigmoidal function pre- and post-meal for meal-matched (A) and non-matched (B) odor pairs. (C) There was no significant change in the sigmoidal slope from before to after the meal for the meal-matched odor pair (t(29) = 1.46, p = 0.15) or the non-matched odor pair (t(29) = 0.42, p = 0.68). Error bars depict within-subject SEM for n = 30. Individual participant data summarized in these plots can be found in S1 Data. SEM, standard error of the mean. (TIF) [file pbio.3001374.s005.tif]

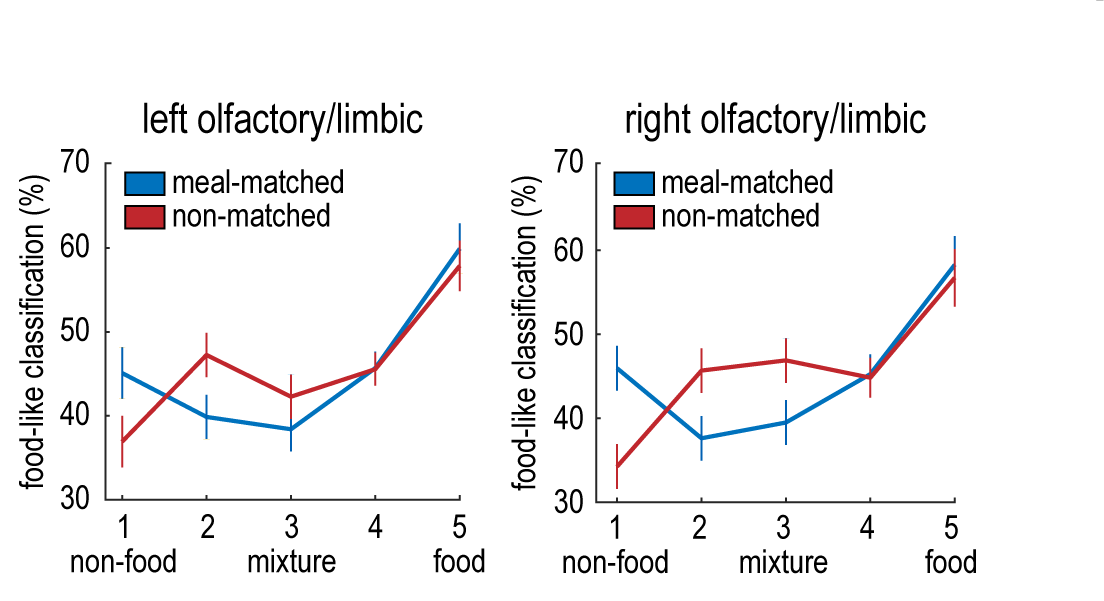

Supplement: S6 Fig — The SVM classifier identified fMRI patterns as food-like less often for meal-matched mixtures than non-matched mixtures in left and right olfactory/limbic ROIs (Fig 5). When considering food-like classification values for each odor mixture separately in these ROIs, differences seem to be driven largely by the 2 mixtures closer to the nonfood side of the spectrum. Note that the large difference in classification is expected for the pure odor endpoints given that ROIs were defined based on ability to discriminate pure food vs. nonfood odors. Individual participant data summarized in these plots can be found in S1 Data. fMRI, functional magnetic resonance imaging; ROI, region of interest; SVM, support vector machine. (TIF) [file pbio.3001374.s006.tif]

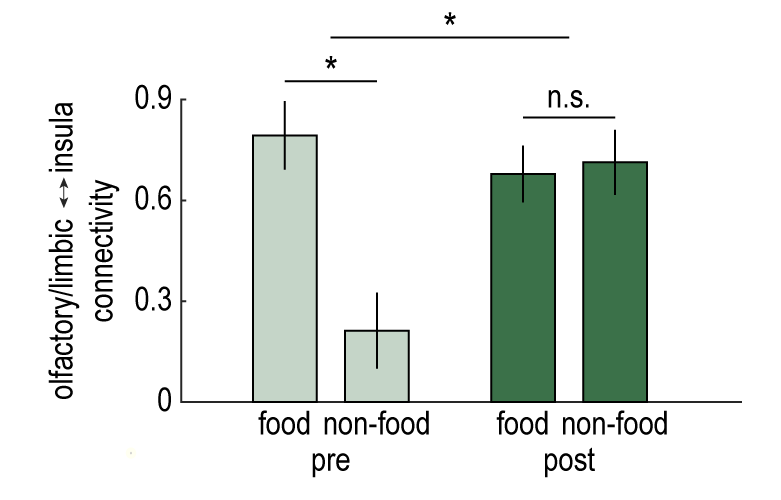

Supplement: S7 Fig — Connectivity differences between food and nonfood odor trials were significant pre-meal (t(29) = 3.33, p = 0.002), but not post-meal (t(29) = 0.26, p = 0.79; odor by session interaction, t(29) = 2.62, p = 0.01). This interaction was driven by an increase in nonfood odor connectivity from pre- to post-meal, while connectivity in response to food odors did not change. Error bars depict within-subject SEM for n = 30. Individual participant data summarized in these plots can be found in S1 Data. SEM, standard error of the mean. (TIF) [file pbio.3001374.s007.tif]
