# Supplementary material for: Olfactory perceptual decision-making is biased by motivational state
Source: PLoS Biol. 2021 Aug 26;19(8):e3001374. doi: 10.1371/journal.pbio.3001374 (PMC8389475; doi:10.1371/journal.pbio.3001374)
Supplement: S1 Table — The group-level t-map was thresholded at pFWE < 0.05 with a minimum cluster size of 15 voxels. Individual participant data summarized in these plots can be found in S1 Data. fMRI, functional magnetic resonance imaging. (DOCX) [file pbio.3001374.s008.docx]

| **Brain region** | **MNI coordinate (x, y, z)** | **Cluster size** | ***T-value*** |
| --- | --- | --- | --- |
| L olfactory/limbic | -28, -6, -18 | 307 | 6.81 |
| R olfactory/limbic | 20, -8, -18 | 373 | 10.11 |
| L mid insula (BA 48) | -40, 0, -4 | 63 | 6.38 |
| R mid insula (BA 48) | 40, -2, 2 | 206 | 7.85 |
| L mid insula (BA 48) | -58, -2, 12 | 46 | 5.77 |
| R mid insula (BA 48) | 60, -12, 16 | 105 | 6.29 |
| R precentral gyrus (BA 6) | 46, 0, 48 | 17 | 5.80 |
| R middle frontal gyrus (BA 6) | 40, -6, 58 | 20 | 6.09 |
| L inferior temporal gyrus (BA 37) | -48, -52, -18 | 23 | 6.04 |
| R middle temporal gyrus (BA 21) | 66, -12, -12 | 116 | 6.83 |
| L temporal pole (BA 20) | -28, 22, -40 | 38 | 6.58 |
| L supramarginal gyrus (BA 40) | -60, -42, 36 | 59 | 6.27 |
| R angular gyrus (BA 7) | 30, -60, 46 | 21 | 6.05 |
